# What individual, family, and school factors influence the identification of special educational needs in Wales?

**DOI:** 10.1111/bjep.12760

**Published:** 2025-03-03

**Authors:** Jennifer Keating, Cathryn Knight, Alexandra Sandu, Robert French

**Affiliations:** ^1^ Administrative Data Research Wales Cardiff University Cardiff UK; ^2^ Wales Institute of Social and Economic Research and Data Cardiff University Cardiff UK; ^3^ School of Education University of Bristol Bristol UK

**Keywords:** additional learning needs, administrative data, SEN identification, special educational needs, Wales

## Abstract

**Background:**

Previous national and international research has investigated potential patterns of SEN identification, in which there may be overrepresentation of males, individuals from lower socio‐economic backgrounds, and pupils attending schools in economically disadvantaged areas.

**Aims:**

The aim of the current study is to link administrative education data for the academic year 2011/12 to data from the UK 2011 Census to explore which individual, family and school characteristics are associated with SEN identification.

**Sample(s):**

The analysis sample consists of 284,010 pupils attending schools in Wales in 2011/12 linked to household data from the UK 2011 Census.

**Methods:**

Multilevel models were used to estimate the association between individual, family and school characteristics with SEN identification. Further models examined how these factors influence four areas of SEN needs: cognition and learning; communication and interaction; physical and/or sensory; and behavioural, emotional and social development.

**Results:**

Results suggest that aspects of a child's individual and family environment are associated with SEN identification. In particular, males, pupils reported as White ethnicity, pupils who were persistently absent, pupils from households with lower parental education, parental economic inactivity, and lower household social grades have an increased likelihood of SEN identification.

**Conclusions:**

This study emphasizes the importance of considering the environmental context (family and school) of the child in addition to child characteristics for a more accurate and holistic understanding of a child's needs. This research can inform the development of more inclusive and effective support strategies under the new Additional Learning Needs framework in Wales.

## INTRODUCTION

A large and growing body of literature has reported that various factors in a child's environment can influence whether they will be identified with special educational needs (SEN) or will require educational support. From a bio‐ecological perspective (e.g. Bronfenbrenner, 1977, 2005), children's development is shaped through bidirectional interactions at the micro, meso, and macro system levels. These layers of influence include parents, siblings, teachers and the wider school context. At the macro level, administrative data from England found that children's socio‐economic background was a significant predictor of SEN identification (Hutchinson et al., 2021; Strand & Lindorff, 2021; Strand & Lindsay, 2009). Meso level influences have been found by Hutchinson et al. (2021) who identified that the school a child attended was a significant predictor of SEN identification, with the variance attributable to individual characteristics reducing by half once school factors were accounted for. In addition, ethnic disparities have been reported in English administrative data (Strand & Lindorff, 2021), although these findings were often explained by socio‐economic demographics and prior attainment. A recent study using administrative data from Wales also supports the influence of socio‐economic factors in SEN identification but finds less influence of schools (Knight et al., 2024). This article offers a novel insight into these layers of influence in a Welsh context by combining Census data with administrative education data.

It is therefore important to understand whether there are different patterns of SEN identification across individuals, micro, meso and macro levels in order to identify and begin to address the reasons for any inequalities in SEN identification. By understanding where differences in SEN identification and subsequent support may exist, we can develop the appropriate targeted support to ensure that all children receive appropriate, inclusive, effective education (Hornby, 2011). Understanding these patterns of identification is particularly pertinent in the current context of the Welsh education system. Wales is currently transitioning to a new additional learning needs (ALN) provision system, a unified system for 0–25‐year‐olds (Welsh Government, 2018). The current study uses administrative data from the older SEN system in order to provide an important baseline to understand how the new ALN system may improve education support for children in Wales. Due to the data coming from the former SEN system, we use the term SEN throughout.

The previous SEN Code of Practice in Wales (2004) defined a child as having special educational needs ‘if they have a learning difficulty which calls for special educational provision to be made for them’ (p1). Under the SEN system, children could receive one of three levels of provision, depending on the intensity of support required: School Action, School Action+, or Statement. School Action involved initial support for mainstream students, including tailored interventions (e.g. individual help for maths/literacy from a teaching assistant). School Action+ was a more intensive strand, with specialized support and collaboration with external specialists (e.g. support from a speech and language therapist). The Statement system consisted of formal assessment and a legal document outlining the child's needs and necessary provisions which would be managed by the local authority. Previous research suggests that having additional needs is associated with poorer education outcomes such as lower attainment and attendance (e.g. conduct disorder; Dalsgaard et al., 2020; ADHD; Loe & Feldman, 2007). However, a recent study suggested that receiving SEN support could reduce the odds of being absent or excluded from school (John et al., 2022), highlighting the importance of SEN identification for improving outcomes.

Over the last number of years, ~20% of learners in Wales were identified with SEN each year (Senedd Research, 2022; StatsWales, 2024a). However, this number has declined significantly since the introduction of the new ALN system, reaching 11.2% in January 2024 (Welsh Government, 2024). Similar numbers of students receive Special Educational Needs and Disability (SEND) support across England (13.6%; Department of Education, 2024). Although higher rates are seen in Additional Support Needs (ASN) identification in Scotland (37%; Scottish Government, 2023) and SEN identification in Northern Ireland (18.2%; O'Connor et al., 2023) due to each country taking different approaches to defining and supporting SEN (Knight et al., 2023). Given this drastic fall in identification under the new ALN system in Wales, it is important to first establish how children are likely to be identified under the previous SEN system, in order to be able to then consider how this may have changed within the new ALN system.

Children may receive SEN support for having a range of neurodevelopmental conditions (e.g. ADHD, autism, dyslexia), physical conditions (e.g. visual impairment, hearing impairment) or acquired conditions (i.e. caused by injury). The ALN Code for Wales (Welsh Government, 2021) identified four broad areas of need that children may have: communication and interaction, cognition and learning, physical and/or sensory needs, and behaviour, emotional and social development. Previous research has suggested that different factors may influence different types of SEN. Physical difficulties (e.g. visual impairment) are often more objectively identified compared with mental health difficulties, which may be influenced by family circumstances, school resources or teacher recognition (McCoy et al., 2012; van der Veen et al., 2010). For example, Parsons and Platt (2013) reported that children with learning, behaviour and speech difficulties had the most socio‐economic disadvantage of all SEN types. Thus, the pattern of SEN identification may differ based on the identified area of need a child has.

### Individual characteristics

One key factor influencing the identification of children with SEN is gender. Previous research shows that males are more likely to be identified for special education classes (Anderson et al., 2015; Mann et al., 2007; Smeets & Roeleveld, 2016), social and emotional behavioural difficulties (Banks et al., 2012; McCoy et al., 2012; Ormiston & Renshaw, 2023; Strand & Lindsay, 2009), speech impairment (McCoy et al., 2012), learning difficulties (McCoy et al., 2012; Strand & Lindsay, 2009), reading difficulties (Anders et al., 2011), and autism (King & Bearman, 2011) than females.

Race and ethnicity are also factors influencing SEN identification. For example, Cruz et al. (2021) found that African American and Latinx students were more likely to have individual education plans (IEP) than White students, while Asian students were less likely in an American context. Similarly, Anderson et al. (2015) reported that Asian and Hispanic students were less likely than White students to be identified for special education. In the UK context, Strand and Lindorff (2021) found that Black Caribbean and Pakistani pupils in English schools were more likely to be identified with mild learning disabilities, although this was due to socio‐economic disadvantage rather than school identification processes. When controlling for SES and prior attainment, most ethnic minority groups (including Indian, Bangladeshi, Chinese and Black African) were half as likely as White British pupils to be identified with mild learning disabilities (Strand & Lindorff, 2021). However, they found that Black Caribbean and mixed White and Black Caribbean (MWBC) pupils were more likely to be identified with social, emotional and mental health difficulties, even after controlling for demographic characteristics (Strand & Lindorff, 2021). Sullivan (2013) also found that White pupils were more likely to be identified with autism than Black, Hispanic and American Indian/Alaskan Native students, although they were less likely than Asian/Pacific Islander students to be identified with autism.

### Family characteristics

A number of family characteristics have been identified in previous research which may influence SEN identification: parental education, household income, parent occupation, parent separation, number of people in the household and birth order.

Several studies have reported that lower parent education attainment increases the chances of children being identified with SEN. This was found for a range of conditions, such as children having major depressive disorder (for paternal education only; Koch et al., 2022), ADHD (Keilow et al., 2020; Torvik et al., 2020), depression (Torvik et al., 2020; Zhou et al., 2018 [maternal education only]), reading difficulties (Anders et al., 2011; Ehrhardt et al., 2013; Jackson, 2003), calculation difficulties (Anders et al., 2011; Jackson, 2003), learning difficulties (Margai & Henry, 2003; McCoy et al., 2012), behavioural problems (Jackson, 2003; McCoy et al., 2012), anxiety (Ozer et al., 2008), emotional and behavioural difficulties (Stoutjesdijk et al., 2012) and general SEN classification at age 5 (Smeets & Roeleveld, 2016). McCoy et al. (2012) also reported an increased risk of being identified with multiple SEN conditions when parents' education attainment was low.

In contrast, some studies identified that higher parent education was associated with an increased risk of SEN identification in children. This was particularly found for autism (Hsu et al., 2023; King & Bearman, 2011; Maenner et al., 2009). Higher paternal education was also associated with an increased risk of internalizing disorders (Tomić et al., 2012).

Another factor commonly reported is the effect of household income on children's SEN identification. Across a variety of conditions, studies showed that lower household income increased the risk of children being identified with SEN: autism (Hsu et al., 2023), depression (Demkowicz et al., 2021; Zhou et al., 2018), reading difficulties (Ehrhardt et al., 2013), learning difficulties (Margai & Henry, 2003), emotional and behavioural difficulties (Banks et al., 2012), general special education (Anderson et al., 2015; Mann et al., 2007) and anxiety (Demkowicz et al., 2021). Studies that used receiving free or reduced‐price lunch as a proxy for low income also found an increased risk of social and emotional difficulties (Ormiston & Renshaw, 2023; Strand & Lindsay, 2009) and moderate learning difficulties (Strand & Lindsay, 2009). Conversely, two studies found that higher income was associated with higher rates of autism (King & Bearman, 2011; Maenner et al., 2009). This follows a similar pattern of findings that higher parent education was associated with an increase in autism. A further study identified that higher SES was associated with a greater likelihood of special education (Oswald et al., 2006). On the other hand, lower paternal income was associated with a decrease in broad anorexia nervosa (Koch et al., 2022).

In terms of parental occupation, unemployment was associated with an increased risk of ADHD identification (Keilow et al., 2020) and being identified with learning difficulties, emotional and behavioural difficulties, and multiple conditions (McCoy et al., 2012).

The separation of parents or living in a one‐parent household was associated with depression (Srivastava et al., 2019), being in specialist education (Mann et al., 2007), or having increased rates of emotional and behavioural difficulties (Banks et al., 2012). The number of individuals in a household has also been found to play a role in SEN identification. For example, having more children in the family increased the chances of an externalizing disorder; however, more adults in the household acted as a protective factor against externalizing disorders (Tomić et al., 2012). Being in a larger family has also been associated with increased anxiety (Ozer et al., 2008) and emotional behavioural difficulties (Khamis, 2009). One study reported that having more siblings lowered the risk of having an eating disorder or major depressive disorder compared with not having siblings (Koch et al., 2022). Taken together, this previous research shows how wider family factors can play a role in SEN identification. However, these factors have not been explored previously in Wales.

### School characteristics

Several school‐level characteristics have been identified to influence children's likelihood of being identified with SEN. Oldfield et al. (2017) identified that as secondary school size increased, there was a resulting increase in behavioural difficulties. When attempting to discriminate among special educational classroom sizes (by level of need), school climate and teacher effectiveness were the best discriminators among classroom groups (Schwartz & Others, 1988). Larger schools were also associated with higher teacher ratings of internalizing and externalizing behaviours (Peters, 2010).

For children with profound learning difficulties, the economic level of the school district also discriminated among special classroom groups (Schwartz & Others, 1988). The location of the school (urban/rural) also played a role. McCoy et al. (2012) identified that children were more likely to be identified with physical conditions if they attended rural schools in disadvantaged areas. In addition, learning difficulties were more likely to be identified in co‐education and girls‐only schools, but less likely in disadvantaged urban areas. Schools in disadvantaged areas were also more likely to identify emotional and behavioural difficulties (McCoy et al., 2012). Collectively, these studies outline that school factors can also play a role in children's identification with SEN, which has not yet been examined in the Welsh context.

### The current study

The aim of the current study was to identify which individual, family and school factors influence the identification of SEN using a population‐level administrative education dataset from Wales, acknowledging the bio‐ecological systems in which a child exists. The majority of studies mentioned previously have small sample sizes and/or focus on specific age ranges or school year groups. We build on previous research in Wales by linking data on family information from the United Kingdom 2011 Census to administrative education data from the Welsh Government for the academic year 2011/12 along with including information about school characteristics. This enables us to consider individual, household, and school‐level factors in a sample of 284,010 pupils spanning all school years (Reception to Year 11). Further, this allows additional analysis by area of need to examine how these factors may differ across different SEN types. As such, this study asks:

1. What individual, family and school characteristics predict SEN identification?
2. What individual, family and school characteristics predict the identification of the following SEN areas of need:
  - Behaviour, emotional and social development?
  - Cognition and learning difficulties?
  - Physical and/or sensory needs?
  - Communication and interaction difficulties?

## MATERIALS AND METHODS

### Participants

The data in the current study comprises administrative education data from the Welsh Government for the academic year 2011/2012 (Huxley et al., 2025) and the 2011 Census. The 2011/12 academic year was chosen for the following reasons: (1) it closely aligned with the timing of data collection for the 2011 Census, (2) the list of SEN types schools could select in the Pupil Level Annual School Census (PLASC; SEN information source for the current study) was expanded beginning in 2012. This administrative education dataset captures all students enrolled in state‐funded mainstream schools in Wales who could be linked to their corresponding household data from the 2011 Census. Data access approval was granted by the Secure Anonymized Information Linkage (SAIL) Information Governance Review Panel, and ethical approval for the study was granted by the University Ethics Committee.

### Procedures

**FIGURE 1:**
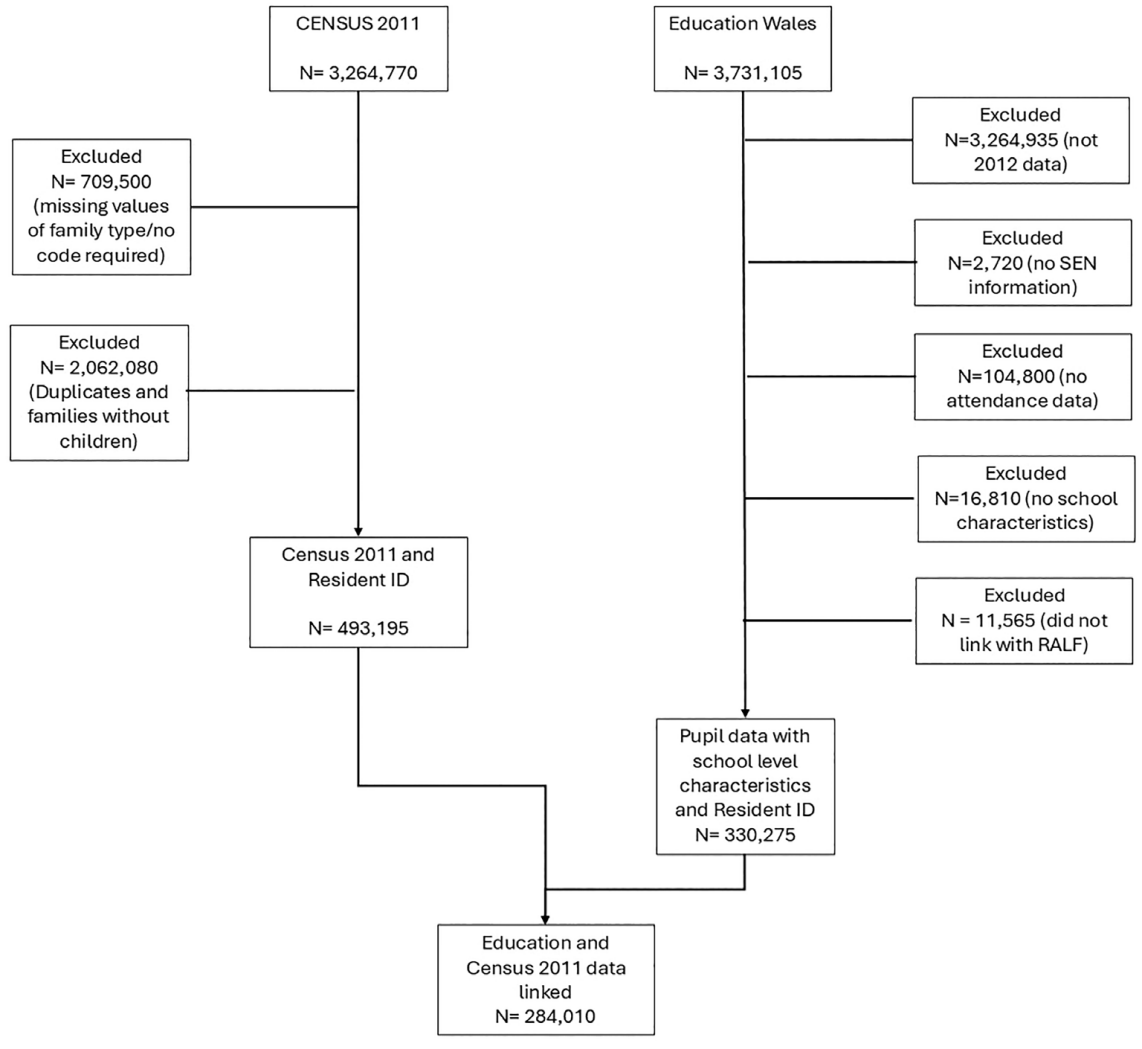
Consort diagram showing data linkage process.

The following education datasets were accessed from the SAIL databank (Ford et al., 2009; Jones et al., 2014; Lyons et al., 2009; Rodgers et al., 2009, 2012, [htps://saildatabank.com]): the Welsh Government Education Dataset, which includes records for all pupils registered at mainstream state schools in Wales, and the 2011 UK Census, a record of all households in the UK on 27th March 2011. The education dataset contains information on pupil demographics (gender, ethnicity), attendance, whether a child is receiving SEN support, and school demographics (number of pupils, number of teachers, school SES). Each individual within the datasets is assigned a unique anonymized linking field (ALF) that replaces any identifiable information. Similarly, each school is assigned a unique school ID. This ALF is used to link each pupil's information across the education datasets, and pupils were then linked to their respective school's demographics by the school ID. The combined pupil and school data were then linked to the Residential Anonymized Linking Field (RALF) data in order to link to Census data. The Census 2011 data were separately linked to the RALF data, before then being linked to education data via RALF. Figure 1 demonstrates how this linking process works. The final sample after linking was 284,010 pupils across Reception year (first year of primary school, 5–6 years of age) to Year 11 (final year of compulsive schooling, 15–16 years of age) for the academic year 2011/12 in Wales.

### Variables

#### Outcome variable

SEN Status: SEN status extracted from the SEN education dataset according to the Pupil Level Annual School Census (PLASC). SEN status is a binary variable, with 1 indicating that the pupil was receiving SEN provision in the 2011/12 academic year and 0 indicating that the student was not receiving SEN provision in the 2011/12 academic year. In the 2011/12 academic year, 23.75% of students received SEN provision. SEN status was used as a binary outcome in each multilevel model.

#### Individual characteristics

SEN Area of Need: In the SEN education dataset, a school could list up to two SEN Types for each child from a total of 17 categories (e.g. ADHD, visual impairment). These SEN types were used to categorize children according to the ALN Broad Areas of Need (Welsh Government, 2021): behaviour, emotional, and social development; communication and interaction difficulties; cognition and learning difficulties; and physical and/or sensory needs. Children with ADHD or behavioural, emotional and social difficulties were categorized with behavioural, emotional and social development needs. Autistic children and those with speech, language and communication difficulties were categorized with communication and interaction needs. Those with dyscalculia, dyslexia, dyspraxia, general learning difficulties, mild learning difficulties, profound and multiple learning difficulties, severe learning difficulties or specific learning difficulties were categorized as having cognition and learning difficulties. Finally, children with hearing impairment, multisensory impairment, physical and medical difficulties or visual impairment were categorized with physical and/or sensory needs. If children were listed to have two SEN types, they were categorized as having multiple difficulties. Children with 2 SEN types often had SEN types across different areas of need, and so we felt it was appropriate to consider these children as a separate group with more complex needs. The number of pupils categorized with each area of need is presented in Table 3.

Gender: the school reported the gender of each student in PLASC. In 2011/12 data, only the binary male and female options were provided.

Ethnicity: the school also reported the ethnicity of each student in PLASC. The PLASC contains 93 possible ethnic codes. In the current study, these were grouped into 5 categories: White, Black, Asian, Mixed, Other.

Attendance: attendance data are provided annually and consists of the number of sessions a child attended during the school year, the number of sessions missed, and the number of possible sessions. The percentage of sessions missed was calculated (number of sessions missed/number of possible sessions × 100). If children missed >10% of sessions during the year, they were considered to be persistently absent. If children missed <10% of sessions, they were not persistently absent, in accordance with Welsh Government guidelines (Welsh Government, 2023).

#### Family characteristics

Highest household qualification: Information on qualifications was provided by the 2011 Census. Each adult was required to list their highest qualification, and this was categorized on a scale from 0 (no qualification) to 3 (university degree). The highest qualification listed for each household was used in the current study.

Household economic activity: information on whether adults in the household were economically active was obtained from the 2011 Census. A binary variable was created to indicate if at least one adult in the household was economically active (1) or not (0).

Family type: the 2011 Census also provided information on family composition. A binary variable was created to identify if children lived in a single‐parent household (0) or dual‐parent household (1).

Number of children in the household: the 2011 Census provided information on the number of dependent children in the household. This was categorized as ‘one dependent child’, ‘two dependent children’, or ‘three or more dependent children’.

Household social class: social grade is a socio‐economic classification used by the Market Research and Marketing industries that is obtained from the 2011 Census. An approximated social grade is calculated for each adult in the household aged 16–64 based on other census data such as qualifications, employment status, occupation and tenure. It consists of four categories: AB (higher and intermediate managerial, administrative and professional occupations), C1 (supervisory, clerical and junior managerial, administrative and professional occupations), C2 (skilled manual occupations), D (semi‐skilled and unskilled manual occupations, and lowest grade occupations) and E (non‐working). The highest social class for any individual in the household was used as the household social class in the current study.

#### School characteristics

School language medium: the PLASC provided information on the language medium of each school. Schools could be classed in one of four categories: bilingual (using both English and Welsh), English medium, Welsh medium, or transitional (a school transitioning from English medium to Welsh medium).

School SES: School SES is measured based on the Wales Index of Multiple Deprivation (WIMD) (Welsh Government, 2019). Information on the WIMD quintile of each school is provided in the education datasets.

Teacher pupil ratio: A teacher pupil ratio was calculated by dividing the number of pupils by the number of teachers in each school.

### Statistical analysis

Data were retrieved from the SAIL databank using SQL. Any counts/frequencies are rounded to the nearest 5 in accordance with disclosure protocols from the SAIL databank and the Higher Education Statistics Agency (HESA). All statistical analyses were done in R (version 4.3.3; R Core Team, 2021), accessed through RStudio (24.04.0). To investigate the influence that individual, family and school characteristics had on the outcome of a child being identified with SEN, multilevel models were used to estimate the odds for each covariate, while accounting for the fact that children are nested within schools, which are nested in local authorities (Rasbash et al., 2010). Multilevel logistic regression models were estimated using the glmmTMB package (Brooks et al., 2017).

The first multilevel logistic regression explored the individual, family and school factors influencing SEN status across all school years and all SEN types. To address the second research question, five subsets of the data were created to include children with each area of need and a comparison group of children not identified with SEN. The same multilevel model was then estimated using each of these five datasets to provide estimates across the different areas of need. Odds ratios and confidence intervals were then calculated for each model using the broom.mixed package (Bolker & Robinson, 2022).

Along with the above analysis, we also ran a single‐level multinomial logistic regression to explore how individual, family and school factors differ across the different levels of SEN: School Action, School Action Plus, Statement. Furthermore, we ran a single‐level multinomial logistic regression including all SEN areas of need in a single model. The results for both models show very similar patterns to the models described above. Therefore, in the interest of brevity, we have opted not to report them here.

## RESULTS

### Descriptives

**FIGURE 2:**
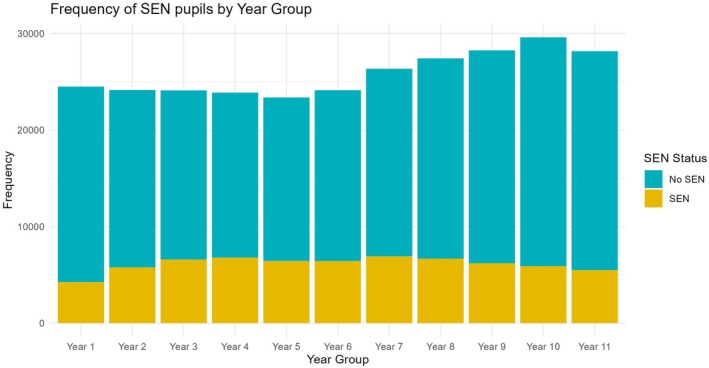
Number of pupils with and without SEN in each year group.

The data linkage process resulted in a sample of 284,010 children with both education and Census data, including one cohort from each year of compulsory schooling. The number of pupils in each year group both with and without SEN across the dataset are presented in Figure 2; the spread is even across years, though we note only 30 children from Reception year were included (due to low linkage with attendance data). The individual, family and school demographics are presented in Table 1.

**TABLE 1:** Sample demographics (*n* = 284,010).

Variable	Category	Frequency
**Individual‐level**		
Gender	Male	145,440 (51%)
	Female	138,570 (49%)
Ethnicity	White	266,260 (94%)
	Asian	5635 (2%)
	Black	1600 (1%)
	Mixed	5760 (2%)
	Other	2430 (1%)
	Unknown	2325 (1%)
Attendance	Not persistently absent	227,730 (80%)
	Persistently absent	56,200 (20%)
	Missing	80 (<.01%)
SEN	No SEN	216,540 (76%)
	SEN	67,475 (24%)
**Family‐level**		
Highest qualification in household	Degree	106,500 (37%)
	Apprenticeship/A Levels	66,205 (23%)
	GCSE	84,525 (30%)
	No qualification	26,780 (9%)
Household economic activity	Economically active	248,425 (87%)
	Economically inactive	35,585 (13%)
Number of parents in household	Not lone parent household	210,765 (74%)
	Lone parent household	73,245 (26%)
Number of children in household	One dependent child	58,215 (20%)
	Two dependent children	133, 205 (47%)
	Three or more dependent children	92,590 (33%)
Household social class	Higher (AB)	53,145 (19%)
	Supervisory (C1)	76,765 (27%)
	Skilled (C2)	72,085 (25%)
	Semi‐skilled (D)	68,445 (24%)
	Not working (E)	13,575 (5%)
**School‐level**		
School language medium	English	945 (63%)
	Bilingual	55 (4%)
	Transitional	10 (.4%)
	Welsh	420 (28%)
	Missing	75 (5%)
School SES Decile	20% least deprived	200 (13%)
	60%–80% most deprived	355 (24%)
	40%–60% most deprived	380 (25%)
	20%–40% most deprived	315 (21%)
	20% most deprived	260 (17%)
**Teacher pupil ratio**	Mean = 17.75	SD = 3.64

*Note*: Social grade AB = higher and intermediate managerial, administrative, and professional occupations, C1 (supervisory, clerical, and junior managerial, administrative, and professional occupations), C2 (skilled manual occupations), D (semi‐skilled and unskilled manual occupations, and lowest grade occupations) and E (not working). School SES Quintiles are calculated from the Wales Index of Multiple Deprivation (WIMD) for schools. Any counts/frequencies are rounded to the nearest 5 in accordance with disclosure protocols from SAIL databank and the Higher Education Statistics Agency (HESA).

Table 2 shows the results of the multilevel model predicting SEN status. In terms of individual characteristics, the odds of identification for females are 53% lower than the odds of males being identified with SEN. Children from Black, Asian, Mixed and ‘Other’ ethnicities are less likely to be identified with SEN than White children, with odds ratios ranging from .63 to .77. Children who are persistently absent from school (missing >10% of sessions) have 1.5 times higher odds of being identified with SEN compared with children who are not persistently absent. In terms of household characteristics, children are more likely to be identified with SEN if they live in households with a parental qualification below a university degree. This ranges from increased odds of 1.23 if the highest household qualification is an apprenticeship or A‐Levels, to 1.90 if parents have no qualification, compared with households where the highest qualification is a university degree. The odds for those in households who are economically inactive are also 1.35 times higher to have children identified with SEN compared with households who are economically active. Having two children in the household results in a small decrease in the odds of being identified with SEN (OR = .92), while having three children in the household results in a slight increase in odds (OR = 1.11), compared with households with one child. Children from households with a lower social grade than the reference Highest category (AB) category are more likely to be identified with SEN, ranging from an odds ratio of 1.27 for the supervisory category (C1) to 2.05 for the not working category (E). School factors of language medium and school SES have modest influence on SEN status – only those from the most deprived schools are more likely to be identified with SEN than children in the 20% least deprived schools (OR = 1.24). A change from 17.75 pupils per teacher to 21.39 pupils per teacher results in a 1% decrease in the likelihood of being identified with SEN (Mean = 17.75, SD = 3.64). The school a child was in explained 6% of the variance in SEN identification, while the local authority explained 1%.

**TABLE 2:** Multilevel model of individual, school, and family‐level factors predicting SEN identification (ref = not receiving SEN support).

Covariate	Category	Odds ratio (OR)	Standard error	*p*	95% CI	
	Intercept	.27***	.02	<.001	.22	.32
Gender	Male (ref)					
	Female	.47***	.00	<.001	.46	.48
Ethnicity	White (ref)					
	Asian	.63***	.03	<.001	.58	.68
	Black	.77***	.05	<.001	.67	.89
	Mixed	.77***	.03	<.001	.71	.83
	Other	.65***	.04	<.001	.58	.74
	Unknown	1.03	.06	.660	.92	1.15
Attendance	Not persistently absent (ref)					
	Persistently absent	1.55***	.02	<.001	1.51	1.59
Highest qualification in household	Degree (ref)					
	Apprenticeship/A Levels	1.23***	.02	<.001	1.20	1.27
	GCSE	1.38***	.02	<.001	1.34	1.43
	No qualification	1.90***	.04	<.001	1.82	1.98
Household economic activity	Economically active (ref)					
	Economically inactive	1.35***	.02	<.001	1.31	1.40
Number of parents in household	Not lone parent household (ref)					
	Lone parent household	1.12***	.01	<.001	1.09	1.15
Number of children in household	One dependent child (ref)					
	Two dependent children	.92***	.01	<.001	.89	.94
	Three dependent children	1.11***	.02	<.001	1.08	1.14
Household social class	Higher (AB) (ref)					
	Supervisory (C1)	1.27***	.02	<.001	1.22	1.32
	Skilled (C2)	1.49***	.03	<.001	1.43	1.55
	Semi‐skilled (D)	1.70***	.03	<.001	1.64	1.77
	Not working (E)	2.05***	.06	<.001	1.94	2.17
School language medium	English (ref)					
	Bilingual	1.03	.08	.674	.89	1.19
	Transitional	1.38	.30	.143	.90	2.10
	Welsh	.94	.04	.134	.87	1.02
School SES	20% least deprived (ref)					
	60%–80% most deprived	.94	.04	.813	.92	1.11
	40%–60% most deprived	1.06	.05	.257	.96	1.16
	20%–40% most deprived	1.07	.05	.171	.97	1.18
	20% most deprived	1.24***	.06	<.001	1.13	1.37
Teacher pupil ratio	Continuous	.99***	.00	<.001	.98	.99
**Random part**		**Variance**		**ICC**		
Between school variance		.20		.06		
Between LA variance		.04		.01		
Number of schools		1429				
Number of local authorities		22				
Number of learners		230,915				

*p < .05, **p < .01, ***p < .001.

The series of models presented in Table 3 explores how individual, family and school‐level factors influence the identification of different areas of SEN (Table 4).

**TABLE 3:** Multilevel logistic regression of the factors associated with being identified with different types of SEN (ref = never identified with SEN).

Covariate	Category	Learning	Communication	BESD	Physical	Multiple
		Odds Ratio (SE)	Odds Ratio (SE)	Odds Ratio (SE)	Odds Ratio (SE)	Odds Ratio (SE)
	Intercept	.11 (.01) ***	.02 (.00) ***	.03 (.01) ***	.01 (.00) ***	.06 (.01) ***
Gender	Male (ref)					
	Female	.57 (.01) ***	.32 (.01) ***	.29 (.01) ***	.71 (.03) ***	.33 (.01) ***
Ethnicity	White (ref)					
	Asian	.66 (.04) ***	.81 (.09)	.41 (.06) ***	.98 (.14)	.46 (.05) ***
	Black	.76 (.07) **	.88 (.17)	.66 (.13) *	.88 (.25)	.79 (.11)
	Mixed	.75 (.04) ***	1.01 (.10)	.82 (.08)	.72 (.11)	.69 (.06) ***
	Other	.59 (.05) ***	1.08 (.15)	.38 (.09) ***	.80 (.17)	.65 (.09) **
	Unknown	1.04 (.07)	1.01 (.17)	.88 (.15)	1.02 (.22)	1.12 (.13)
Attendance	Not persistently absent (ref)					
	Persistently absent	1.42 (.02) ***	1.30 (.05) ***	1.98 (.06) ***	2.09 (.10) ***	1.73 (.04) ***
Highest qualification in household	Degree (ref)					
	Apprenticeship/A Levels	1.32 (.03) ***	1.07 (.04)	1.17 (.06) **	.96 (.06)	1.24 (.04) ***
	GCSE	1.51 (.03) ***	1.10 (.04) *	1.37 (.06) ***	.98 (.06)	1.38 (.04) ***
	No qualification	2.18 (.06) ***	1.34 (.08) ***	1.70 (.10) ***	.94 (.08)	1.88 (.08) ***
Household economic activity	Economically active (ref)					
	Economically inactive	1.19 (.02) ***	1.35 (.06) ***	1.68 (.07) ***	1.54 (.10) ***	1.75 (.05) ***
Number of parents in household	Not lone parent household (ref)					
	Lone parent household	1.11 (.02) ***	.95 (.03)	1.51 (.05) ***	.85 (.04) **	1.10 (.03) ***
Number of children in household	One dependent child (ref)					
	Two dependent children	.97 (.02)	.92 (.04) *	.77 (.03) ***	.85 (.04) **	.85 (.02) ***
	Three or more dependent children	1.20 (.02) ***	1.07 (.04)	.89 (.04) **	.91 (.05)	1.03 (.03)
Household social class	Higher (AB) (ref)					
	Supervisory (C1)	1.27 (.03) ***	1.23 (.06) ***	1.37 (.08) ***	1.12 (.07)	1.32 (.06) ***
	Skilled (C2)	1.56 (.04) ***	1.33 (.07) ***	1.37 (.09) ***	1.21 (.08) **	1.56 (.07) ***
	Semi‐skilled (D)	1.79 (.05) ***	1.49 (.08) ***	1.72 (.11) ***	1.15 (.08)	1.82 (.08) ***
	Not working (E)	2.11 (.08) ***	1.75 (.14) ***	2.04 (.16) ***	1.10 (.13)	2.36 (.14) ***
School language medium	English (ref)					
	Bilingual	1.00 (.09)	.83 (.14)	.79 (.11)	.85 (.14)	1.00 (.15)
	Transitional	1.31 (.35)	2.22 (1.02)	2.71 (1.08) *	.79 (.46)	1.05 (.46)
	Welsh	1.05 (.05)	.89 (.08)	.82 (.07) *	.82 (.07) *	.64 (.06) ***
School SES	20% least deprived (ref)					
	60%–80% most deprived	.94 (.06)	1.28 (.14) *	1.03 (.10)	1.05 (.11)	. 95 (.10)
	40%–60% most deprived	1.02 (.06)	1.28 (.14) *	1.06 (.10)	.98 (.10)	1.00 (.10)
	20%–40% most deprived	1.04 (.06)	1.20 (.13)	1.06 (.10)	1.01 (.11)	1.00 (.10)
	20% most deprived	1.15 (.07) *	1.53 (.17) ***	1.34 (.13) **	1.15 (.13)	1.24 (.13) *
Teacher pupil ratio	Continuous	.99 (.00)	1.00 (.01)	.97 (.01) ***	1.00 (.01)	.97 (.01) ***
		**Variance (SD)**	**Variance (SD)**	**Variance (SD)**	**Variance (SD)**	**Variance (SD)**
Random part	Between school variance	.30 (.55)	.80 (.90)	.49 (.70)	.46 (.68)	.78 (.88)
	Between LA variance	.07 (.26)	.12 (.35)	.04 (.19)	.03 (.18)	.21 (.46)
	Number of schools	1429	1429	1429	1429	1429
	Number of Local Authorities	22	22	22	22	22
	Number of learners	206,525	180,375	179,845	177,265	185,425
	Number of learners with SEN	38,330	6470	6465	3180	13,000

*Note*: Communication = communication and interaction needs; Learning = cognition and learning difficulties; Multiple = child identified with 2 SEN types; Physical = physical and/or sensory needs.

Abbreviation: BESD = behavioural, emotional, and social development needs.

*p < .05, **p < .01, ***p < .001.

**TABLE 4:** Interclass Correlation Coefficients (ICC) for each model.

ICC	Learning	Communication	BESD	Physical	Multiple
Between school variance	.08	.19	.13	.12	.18
Between LA variance	.02	.03	.01	.01	.05

*Note*: Communication = communication and interaction needs; Learning = cognition and learning difficulties; Multiple = child identified with 2 SEN types; Physical = physical and/or sensory needs.

Abbreviation: BESD = behavioural, emotional, and social development needs.

#### Cognition and learning needs

A similar pattern of findings was observed in the model for cognition and learning needs. The only variables in which results differed from any SEN model were that the odds ratio for children from households with two dependent children and teacher‐pupil ratio were no longer significant. The school a child was in explained 8% of the variance (ICC = .08), while the local authority explained 3% (ICC = .03; Table 4).

#### Communication and interaction needs

For the most part, the results of the model for communication and interaction needs were similar to any SEN. However, no ethnicity was associated with an increased odds of identification with communication and interaction needs, relative to White pupils. Pupils from households with A Levels or apprenticeship as the highest household qualification were not at increased odds relative to pupils from households with a university degree. Children living in a lone parent household were also not at increased odds of being identified with communication and interaction needs. Living in a household with three dependent children did not increase odds, relative to living in a household with one dependent child. Students attending schools in the 60%–80%, 40%–60%, and 20% least deprived schools were more likely to be identified (OR = 1.28, 1.28, and 1.53). A child's school explained 19% of the variance (ICC = .19), while the local authority explained 3% (ICC = .03), although with high standard deviations (Table 4).

#### Behavioural, emotional and social development needs

The model for BESD followed the same pattern as any SEN, except that pupils from mixed ethnic backgrounds no longer showed significantly decreased odds of being identified relative to White pupils. In addition, attending a transitional school was associated with an increase in the odds of being identified with BESD compared with those in an English medium school (OR = 2.71). Children in Welsh language schools were less likely to be identified (OR = .82) than those in English language schools.

The variance explained by schools in this model was 13% (ICC = .13), and 1% of variance was explained by local authorities (ICC = .01; Table 4).

#### Physical and/or sensory needs

The model for physical and/or sensory needs showed the greatest divergence of results from the model for any SEN. No ethnicity was associated with an increased odds of identification, relative to White pupils. There were also no significant associations between household qualifications and identification of physical and/or sensory needs. Living in a household with three dependent children did not increase odds, relative to living in a household with one dependent child. In terms of school factors, learners in Welsh language schools were less likely to be identified than learners in English language schools (OR = .82). No level of school SES was associated with increased odds of identification relative to those in the 20% least deprived schools. The school a child attended explained 12% of variance (ICC = .12), and the local authority explained 1% (ICC = .01; Table 4).

#### Multiple identified needs

The model for multiple needs revealed a similar pattern of results to the model for any SEN, save for some minor variations. Pupils of black ethnicity were not at increased odds of being identified with multiple needs. Children living in households with three or more dependent children did not have increased odds of identification. Although children attending a Welsh language school were less likely to be identified with SEN (OR = .64). The school a child was in explained 18% of the variance (ICC = .18), while the local authority explained 5% (ICC = .05), although these had large standard deviations.

## DISCUSSION

The aim of this study was to explore what individual, family and school characteristics influence the identification of SEN using a nationwide respective cohort of children in Wales, exploring how children's environments shape their development in line with Bronfenbrenner's ecological systems theory (Bronfenbrenner & Morris, 2007). To the bset of our knowledge, this is the first study in Wales to link education and Census data to explore these individual, family, and school‐level factors. Our results show that household characteristics captured by the Census, including parent qualifications, family structure, economic activity, and social grade, influence SEN identification, in addition to individual characteristics. Our results further show that these patterns of association vary across different areas of SEN need.

One key finding was the strong association between family characteristics and SEN identification. The stronger association between lower socio‐economic background and SEN identification aligns with previous research with a different cohort of children in Wales (Knight et al., 2024), in addition to research in the English context (Hutchinson et al., 2021; Parsons & Platt, 2013). For example, Hutchinson et al. (2021) report that a child living in the highest decile area of deprivation has a 97.4% chance of being identified with SEN, compared with 11.5% in the lowest decile area of deprivation. In a 2013 report, Parsons and Platt found that children with SEN were significantly more likely to have less qualified parents to live in a single parent or non‐working household, to live in rented social housing and to experience income poverty. The current study extended previous findings by adding linked Census data to capture household characteristics and examining patterns of identification across SEN areas of need. We found that higher household qualification was associated with lower SEN identification. This gradient persisted for SEN overall, and across the models for each SEN area of need. Looking at patterns by SEN areas of need, the identification of cognition and learning needs were more likely when there were lower levels of parental education in the household. On the other hand, there were weaker associations between household qualifications and identification of communication and interaction needs. Children from economically inactive households were more likely to be identified with SEN in general relative to children from economically active households, with identification most likely for children with BESD and multiple needs. The association between living in a lone‐parent household and SEN identification was largest for pupils with BESD. However, it should be noted that this family structure is only captured as of the date of the 2011 Census and does not reflect the possibility of other family structures and support networks which could not be collected by this administrative data.

A second key finding is that physical and/or sensory conditions were less associated with family characteristics. Unlike other SEN needs, physical and/or sensory needs were not associated with parental qualifications or household social grade. This finding is surprising, as SES has been linked to a wide range of health outcomes, and children from lower SES backgrounds have been found to be more likely to experience delayed growth, inadequate neurobehavioural development in utero, premature birth or disability at birth (Bradley & Corwyn, 2002; Crump, 2020). A study from Chou et al, (2015) the US Department of Health (2000) also found a link between low SES and sensory impairment. In contrast, these findings highlight the significant role the environment plays in identifying other types of needs, emphasizing the potentially subjective nature of SEN identification when there is a ‘hidden’ need that is neither physical nor sensory.

Across all areas of need, females were less likely to be identified with SEN. This is in line with previous research, showcasing an increased prevalence in males for a variety of conditions including BESD (Strand & Lindsay, 2009), ADHD (Willcutt, 2012), speech impairment (McCoy et al., 2012), reading difficulties (Anders et al., 2011), and hearing impairment (Mehra et al., 2009). However, our findings also show wide variation across SEN areas of need. For example, females are only 29% less likely to be identified with a physical and/or sensory need, but 71% less likely to be identified with BESD needs. This further speaks to the potentially subjective nature of SEN identification for needs such as learning or behavioural difficulties, in addition to the gender disparity in assessment tools and diagnostic criteria for neurodevelopmental conditions (e.g. Loomes et al., 2017).

In addition, pupils who were persistently absent were more likely to be identified with SEN. This finding is supported by previous research showing pupils with neurodevelopmental conditions or mental health difficulties were more likely to be persistently absent from school (John et al., 2022). This association was particularly strong for pupils with physical and/or sensory needs in the current study. It is unclear whether having additional educational needs may cause a child to refuse or not attend school, leading to persistent absenteeism, or whether a child is identified as needing additional support because they have missed so much schooling. As such, further research is required to tease apart this relationship. Indeed, the nature of this relationship may vary depending on the needs of a child or the child themselves. Longitudinal research examining attendance levels pre‐ and post‐SEN identification could help elucidate this relationship further.

We also found that some aspects of school characteristics were associated with SEN identification. A standard deviation increase in the teacher–pupil ratio was associated with a small increase in the odds of being identified with SEN overall, or with BESD or multiple SEN types. This may be due to the fact that more pupils attend school in the more densely populated regions in South Wales (StatsWales, 2024a), which are also areas of economic disadvantage (WIMD; Welsh Government, 2019). Further geographical analysis would be needed to examine how this relationship plays out in terms of SEN identification. Pupils attending schools in the most deprived quintile were more likely to be identified with BESD needs, cognition and learning needs, and communication and interaction needs relative to children attending schools in the least deprived quintile. This partially replicates the findings of McCoy et al. (2012), in that children attending schools in disadvantaged areas were more likely to be identified with emotional and behavioural difficulties, and less likely to be identified with learning difficulties. Differences across SEN areas were also found for school language medium. Children were more likely to be identified with BESD if attending a transitional school, although it should be noted that there were only a small number of transitional schools in the analysis. Pupils attending Welsh medium schools were less likely to be identified with multiple SEN. It may be that parents of children with complex needs are less likely to send their children to Welsh medium schools due to the extra demand of communicating through Welsh, particularly if it is not a child's first language (Ware, 2014).

We also find only minor variance explained at the school level. Interclass correlation coefficients showed little variation between schools and local authorities. This is similar to Knight et al. (2024) which also reports only 5% of variance is accounted for by schools and 2% explained by local authorities, and suggests some level of consistency across Wales in how SEN is assessed. These findings are in contrast to Hutchinson et al. (2021), who found 71% of variation in the chances of a child being identified with SEN was explained by the school a child attended in England, with only 1% explained by local authorities. Strand and Lindorff (2018) also found similarly small variation at the local authority level in a study using English national data, ranging from <2% for children with social, emotional and mental health difficulties to 5%–6% for children with moderate learning difficulties. However, they did find greater variation at the school level, at 11%–12% for children with autism spectrum conditions, 13%–15% for children with social, emotional and mental health difficulties, and rising to 22%–26% for children with moderate learning difficulties (Strand & Lindorff, 2018). One explanation for this is that there is less variation in school type and funding structure in Wales compared with England (OECD, 2018), which may lead to similar practices and policies across Welsh schools. However, we find more variation accounted for at the school level when we model each SEN area of need, particularly for communication and interaction needs and multiple needs. This may further speak to the subjective identification process of these areas of need.

It is important to acknowledge that funding allocation models exist in different countries and jurisdictions, which will impact SEN identification and cross‐country comparison. At the time the data in the current study was collected (2011/12), local authorities could fund SEN through two means: (1) a delegated budget provided to schools or (2) funds retained centrally within the local authority or school budget. Data from StatsWales suggests that the gross budgeted expenditure per pupil for SEN was £770 in 2011/12 (National Assembly for Wales, 2015). SEN/ALN expenditure per pupil has been raised to £1306 in Wales by 2024/25 (StatsWales, 2024b), while local authorities in England outline a threshold sum of £6000 per pupil with SEN (Department of Education, 2024). However, recent research reported significant differences in High Needs Block funding among LAs in England (Marsh et al., 2024). Similarly, in Wales, the allocated funds to schools for SEN/ALN from LAs are notional, and schools can determine how to spend their budget. This can create variation in how SEN/ALN funding is spent across Wales, perhaps creating disparities between different socioeconomic areas, and is an avenue for future research. It should also be noted that there is also a risk of disproportionality of identification due to household resources. For example, families with greater resources may be able to access private assessments. However, given that the current study found decreased odds of SEN identification for children from families with fewer resources, this does not seem to be influencing the results.

These results lead us to make a number of recommendations. Firstly, a child's environment should be considered in the identification process. Rather than focusing on the learning need alone, a holistic understanding of a child and their environment is needed to best support their learning. This can include educators being aware of potential patterns in SEN identification to ensure that children from more affluent households are not missed, as well as the possibility that children from less affluent households are mislabelled or overrepresented. Secondly, under the new ALN system, thought needs to be given as to how best to target support. These data show that SEN identification goes beyond the school level, particularly in the Welsh context. This suggests that working with families and communities to support them with adequate resources is just as important in identifying needs.

There are a number of limitations to consider in the context of the study's findings. We did not have access to any health data which meant that we could not consider the influence of birth factors (e.g. prematurity, low birth weight), but also limited the information on children's SEN/ALN. In the PLASC, schools can list up to two SEN/ALN types for each child, a primary and a secondary concern. This does not account for more complex needs a child may have. By including health data in future research, a more detailed picture of children's health could be created, to further understand how SEN/ALN provision supports their academic career. In addition, the SEN category ‘general learning difficulties’ is often used as a general term to catch any children requiring support without a specific diagnosis or who may be on the diagnostic pathway. This ‘general learning difficulties’ term is included within the cognition and learning area of need, which contains the largest percentage of students with SEN (56% of students with SEN had cognition and learning needs in the current study). Of note, this SEN category is no longer used in the new ALN system. Therefore, this research offers a baseline to explore how patterns may change now that this label has been removed. Secondly, the current analysis can reveal patterns of association in the data, but we cannot determine the direction or cause of this relationship. This would require more in‐depth analysis with a greater variety of data methods. Additionally, the Census only captures a snapshot of household information as of the Census date. It does not capture changing economic circumstances or the wider family and support networks a child and their family may have. The data used in the current study is from 2011/12 and does not use the most up‐to‐date Census. This is due to (1) the ongoing transition to the ALN system, making it difficult to capture accurate SEN/ALN data across all school years as different year groups undergo the transition in phases, and (2) extended timelines in the release of the 2021 Census by the Office of National Statistics.

In conclusion, this study extends our previous knowledge by linking education data with Census data to capture both household and school characteristics of SEN identification, in addition to examining these factors across different areas of SEN need. We found that individual and family characteristics are associated with SEN identification in a national cohort of Welsh pupils. School characteristics seem less influential on the identification of SEN. We found that this pattern of findings is largely true across three different areas of need: behavioural, emotional, and social development needs; communication and interaction needs; and cognition and learning needs. The identification of pupils with physical and/or sensory needs seems less influenced by home environment variables, in contrast to previous research. In light of the new ALN system introduced in Wales, this research provides valuable insights into the role environmental factors played in the identification of SEN under the previous system. The findings underscore the significance of taking into account a wide range of environmental influences—such as socio‐economic background, family structure and access to educational resources—when assessing SEN. By emphasizing the interplay between these external factors and a child's learning needs, as theorized by Bronfenbrenner (1977, 2005), the study highlights how environmental considerations are essential for a more accurate and comprehensive understanding of a child's needs. This, in turn, informs the development of more inclusive and effective support strategies under the new ALN framework, ensuring that interventions and support are tailored not only to individual learning challenges but also to the broader context in which they occur.

## AUTHOR CONTRIBUTIONS

**Jennifer Keating:** Conceptualization; investigation; writing – original draft; writing – review and editing; methodology; formal analysis; visualization; data curation. **Cathryn Knight:** Writing – review and editing; conceptualization. **Alexandra Sandu:** Writing – review and editing; formal analysis. **Robert French:** Conceptualization; writing – review and editing; supervision; methodology.

## CONFLICT OF INTEREST STATEMENT

The authors declare no conflicts of interest.

## Data Availability

The data used in this study are available in the SAIL Databank at Swansea University (Swansea, UK) but, as restrictions apply, it is not publicly available. All proposals to use SAIL data are subject to review by an independent Information Governance Review Panel.
